# Design and Validation of Vision-Based Exercise Biofeedback for Tele-Rehabilitation

**DOI:** 10.3390/s23031206

**Published:** 2023-01-20

**Authors:** Ali Barzegar Khanghah, Geoff Fernie, Atena Roshan Fekr

**Affiliations:** 1 KITE Research Institute, Toronto Rehabilitation Institute, University Health Network, 550 University Ave, Toronto, ON M5G 2A2, Canada; 2 Institute of Biomedical Engineering, University of Toronto, 164 College St., Toronto, ON M5S 3G9, Canada; 3 Department of Surgery, University of Toronto, 149 College Street, Toronto, ON M5T 1P5, Canada

**Keywords:** tele-rehabilitation, deep learning, biofeedback, artificial intelligence, 3D model

## Abstract

Tele-rehabilitation has the potential to considerably change the way patients are monitored from their homes during the care process, by providing equitable access without the need to travel to rehab centers or shoulder the high cost of personal in-home services. Developing a tele-rehab platform with the capability of automating exercise guidance is likely to have a significant impact on rehabilitation outcomes. In this paper, a new vision-based biofeedback system is designed and validated to identify the quality of performed exercises. This new system will help patients to refine their movements to get the most out of their plan of care. An open dataset was used, which consisted of data from 30 participants performing nine different exercises. Each exercise was labeled as “Correctly” or “Incorrectly” executed by five clinicians. We used a pre-trained 3D Convolution Neural Network (3D-CNN) to design our biofeedback system. The proposed system achieved average accuracy values of 90.57% ± 9.17% and 83.78% ± 7.63% using 10-Fold and Leave-One-Subject-Out (LOSO) cross validation, respectively. In addition, we obtained average F1-scores of 71.78% ± 5.68% using 10-Fold and 60.64% ± 21.3% using LOSO validation. The proposed 3D-CNN was able to classify the rehabilitation videos and feedback on the quality of exercises to help users modify their movement patterns.

## 1. Introduction

Despite significant medical advances, there are still barriers to accessing healthcare facilities for many people with disabilities. Socioeconomic status across various ethnic spectrums and an increasingly aging population have given rise to the demand for enhanced healthcare [1]. Tele-rehabilitation has the exciting potential of providing equitable access for patients at home without the need to travel to rehab centers or shoulder the high cost of personal in-home services. The potential advantages of tele-rehab systems are not only saving on costs [2], but also increasing patient accessibility, especially for people living in rural and remote areas [3].

Over the last few years, the COVID-19 pandemic has highlighted the need for service transitions from in-person to tele-rehab. A survey conducted in late May 2020 [4] in Ontario indicated that even though a gradual return to in-person care was suggested, there was significant interest in continuing to use virtual services even after in-person visits resume. The studies also showed that tele-rehab can produce the same clinical outcome as traditional care approaches [5]. According to the World Health Organization (WHO), one third of the world’s population are living with a health situation that requires rehabilitation [6]. Patients with various problems, such as cardiac diseases; neurological disorders, e.g., brain injury or cognitive problems; musculoskeletal disorders [7]; and vision impairment [8] can benefit from tele-rehab. Therefore, improving tele-rehab platforms will likely have a notable impact on a significant portion of the world’s population.

Tele-rehab services comprise a wide range of offerings, such as assessment, monitoring, prevention, intervention, supervision, education, consultation, and coaching [9]. These services are often in the form of: (i) live videoconferencing, (ii) asynchronous store and forward, (iii) eConsult, (iv) remote patient monitoring (RPM), and (v) mobile health (mHealth), which is the delivery of medicine, public health, and education using mobile devices [9]. Although there are growing technologies for tele-rehab systems, gaps still exist in the practical service delivery to patients. The current tele-rehab platforms often use video conferencing or web-based communication. Albeit useful, this type of tele-rehab is not an efficient way to help patients perform their rehab exercises independently with confidence. In addition, it is an expensive and impractical use of healthcare resources since it requires clinicians to be virtually present for the entire session. This gap highlights the need for novel automatic tele-rehab platforms with the capability to automate exercise guidance. In this paper, a new approach is proposed to design a biofeedback system capable of identifying the correct and incorrect movements using deep learning.

## 2. Literature Review

Many studies have used machine learning (ML) to design automatic tele-rehab systems. For example, Wearable Inertial Measurement Units (IMUs) provide useful information to detect joint angles, acceleration, and motion patterns [10,11]. Argent et al. [12] applied Decision Tree (DT) and Random Forest (RF) to estimate the hip and knee joint angles, using a single IMU. They used a 3D CODA Mocap as their ground truth. The best result with 14 participants showed an average Root Mean Square Error (RMSE) of 4.81 ± 1.89 across eight rehabilitation exercises. Kim et al. [13] proposed a method to measure the severity of elbow spasticity by analyzing the acceleration and rotation of the elbow using data from eight IMUs mounted on the dorsal side of the affected elbow. DT, RF, Support Vector Machine (SVM), linear discriminant analysis, and MLP were trained on data from 48 participants while performing a passive stretch test. The RF model performed best with an accuracy of 95.4%. Burns [14] evaluated four supervised learning methods: KNN, RF, SVM and a Convolutional Recurrent Neural Network (CRNN) to classify seven different shoulder exercises performed by 20 healthy participants. The main goal of this study was to investigate the feasibility of ML models trained on wrist-worn inertial sensor data for shoulder physiotherapy exercise recognition and monitoring. They used an Apple watch to acquire inertial data. The evaluation was carried out using both 5-Fold and Leave-One-Subject-Out (LOSO) cross validation. The classification accuracy was above 94% for all algorithms using 5-Fold. The highest accuracy rate was achieved by CRNN for both 5-Fold and LOSO with 99.4% and 88.9%, respectively. As expected, LOSO yielded a lower accuracy value for identifying shoulder exercises. These previous studies have some limitations, such as the large number of sensors (e.g., eight IMUs), which affects the usability and cost of the system. Another limitation of using IMUs is that the accuracy degrades over time due to biases, drifts, and random noise; therefore, they need frequent re-calibration [15,16]. In addition, studies have shown that most older adults are not compliant with using this technology and do not want to wear the devices [17].

Another technology for designing tele-rehab systems is vision-based approaches. The majority of these systems use the Kinect sensor, Microsoft HoloLens, and other types of optical systems such as RGB cameras. Mottaghi et al. [18] proposed an automatic assessment system which was validated with data from 44 healthy and 34 patient participants with motor disability. They used a Deep Mixture Density Network (DMDN) on the joints’ position and orientation data extracted from Kinect. A multi-branch convolutional layer, plus a Long Short-Term Memory (LSTM), and a Gaussian Mixture Model (GMM) were used to estimate the exercise performance denoted by a clinician as a score of 1 to 10. The RMSE was used to compare the predicted scores to the clinical scores. The results showed that the DMDN with GMM outperforms the other methods with RMSE of 0.12. Cao et al. proposed a novel hybrid deep network combined with LSTM and a Convolutional Neural Network (CNN) for predicting seven stages of the Brunnstrom scale, which is used by clinicians to assess how well their patients are recovering after stroke [19]. This study used data from 23 participants while performing nine exercises to estimate the Brunnstorm scale scored by a therapist. A mean accuracy of 84.1% was achieved in estimating the clinician’s three-class Brunnstrom stages (III, IV, and V).

Esfahlani et al. [20] used ML algorithms to estimate the treatment stages of multiple sclerosis (MS) patients, using a remote home-based neuro-rehabilitation video game intervention. They also proposed classifying the MS based on the participants movements and the score extracted from the Motor Assessment Scale (MAS). In addition to the video game, they used the data acquired from the Kinect system and a wearable armband. The first objective was to achieve a model that could estimate the three treatment stages, i.e., pre-treatment, post treatment, and four-month follow-up. They trained an SVM model on data from a nine-axis IMU mounted on the arm of the participants. This model achieved an RMSE of 6.1% when compared to the ground truth from two therapists. Their second objective was to classify healthy and MS participants based on movement patterns. They trained both SVM and K-Nearest Neighbors (KNN) classifiers with the upper extremity (arm, forearm, and hand) joints’ kinematic and hand gestures data while the participants were playing 15 video games. SVM achieved an accuracy of 88%, and KNN achieved an accuracy of 91.7% using 5-Fold cross validation.

An ML-based virtual physical therapy (PT) was proposed by Wei et al. in [21] for patients with Parkinson disease (PD). They proposed a two-phase human action recognition to first recognize the exercises performed by 35 patients and then to provide feedback on their movement quality. They used hidden Markov models to detect repetitions of each exercise using Kinect data and to segment sub-actions in each repeat. The proposed method achieved accuracy rates of 97.1%, 97.9%, and 99.4% for classifying each of the three different actions. An SVM classifier was applied to classify correctly and incorrectly executed exercises that were labeled by the clinicians. RF-based models were used for task recommendation, including regress, repeat and progress. They reported average accuracy rates of 93.5% and 90.47% for binary classification (correct/incorrect) and task recommendation, respectively.

Among the solutions that use ML techniques, some benefit from the pre-trained models such as MediaPipe, OpenPose, and MoveNet. These models are capable of tracking body pose/body skeletons by detecting key points (joints of the body). For example, Pham et al. [22] extracted skeleton sequences with the help of MediaPipe from RGB footages captured from an off-the-shelf camera to design an automatic recognition and assessment of physical exercises system. Nine healthy participants were recorded performing three exercises, five times each. For the assessment part, the frame-based and the sequence-based scores were calculated. The frame-based approach only assesses the body in a single frame, while the sequence-based approach investigates a sequence of frames. The frame-based score refers to the joint mobility assessment inspired by Cao et al. in [23], and the sequence-based scores uses Dynamic Time Warping (DTW) to assess multiple exercises. For the action recognition phase, a skeleton-based action recognition Double-Feature Double-Motion Network (DD-Net) [24] was used on the extracted skeletons. The action recognition algorithm achieved an accuracy of 98.33%. For the score assessment, the scores were estimated and reported but not evaluated versus any ground truth.

Yan et al. [25] proposed a rehabilitation action recognition system using OpenPose and a full Convolutional Neural Network (FCN). They extracted the body skeleton of stroke patients from RGB videos provided in the stroke rehabilitation action datasets. The datasets comprised six different types of actions. Features were extracted from OpenPose and used to train a one-dimensional (1D) FCN classifier. The results showed100% accuracy in classifying the activity types.

Albeit useful, all these previous works focused on action recognition and did not provide any feedback to the patient to inform them if they performed the exercises in the correct or incorrect way, and finally, to help them refine their movement patterns to get the most out of their plan of care.

Another vision-based tele-rehab system was presented by Barriga et al. in [26]. They designed four different experiments for detecting static posture and falls. The analysis of the data from one participant showed that (i) neural network models are not affected by the participant’s physical properties, e.g., height; (ii) the accuracy of the model mainly depends on the NN characteristics, e.g., topology; and (iii) there is evidence that the distance of the time-of-flight (ToF) camera (e.g., Kinect) from the participant had a direct impact on the performance of the model. A deep learning model that combined CNN and LSTM (3-layer CNN-LSTM) was trained on RGB data to detect seven different rehabilitation exercises recorded from 20 post-stroke patients by Rahman et al. [27]. They compared the results with both CNN and KNN algorithms. The best results were achieved by the proposed 3-layer CNN-LSTM, which showed 91.3% accuracy.

Yahya et al. [28] conducted a study to compare the performance of ML algorithms in estimating the shoulder angle from an RGB camera versus the Kinect output as the ground truth. A feed-forward NN was used to estimate the shoulder joint coordinates collected from two healthy participants. The comparison of the results provided an RMSE of 9.29° and 5.3° in the sagittal and coronal planes, respectively. Tang et al. [29] developed a multi-stream deep neural network (MDNN) for the egocentric action recognition of eight participants. Since egocentric action recognition is usually sensitive to hand poses, they extended the MDNN by integrating the hand information to enhance the recognition accuracy. The cross-subject action recognition accuracy of the proposed system on the WCVS dataset was 65.67% and 67.04% for with and without hand enhancement, respectively. Table 1 summarizes the reviewed vision-based studies in the field of tele-rehabilitation.

## 3. Materials and Methods

### 3.1. Dataset

In this paper, we used the dataset published by Miron et al. in [31]. This dataset consisted of data from 16 patients (P1–P16) and 14 healthy participants (H1–H14) while performing 9 different rehabilitation gestures. The patients’ age range was between 20 and more than 60 years with an average of 43, and the healthy participants’ age range was between 20 and 39 with an average of 26. There were 11 male and 5 female patients, and 7 male and 7 female healthy participants. The patients were selected from a diverse patient population (5 had a spinal cord injury, 5 were post-stroke patients, 1 had a brain injury and 5 had neurological conditions). The healthy participants were 7 physiotherapists and 7 trainees. The performed gestures are listed in Table 2.

**Figure 1:**
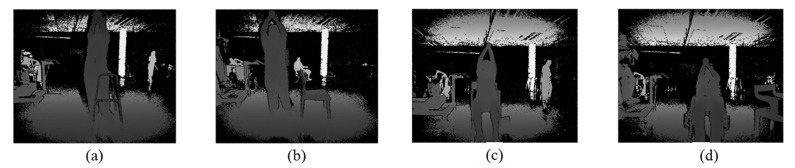
Frames showing patients during SFE gesture in all 4 positions: (**a**) standing using a stand frame, (**b**) standing, (**c**) sitting on a chair, and (**d**) sitting on a wheelchair.

The data were collected by a Microsoft Kinect One sensor. The depth videos and skeleton tracking data for each subject have been made available online [32]. The patients were asked to perform the gestures in the most comfortable position. Five patients sat in a wheelchair, 1 stood using a stand frame, 2 sat on a chair, and 8 stood normally. An example picture of the Shoulder Forward Elevation (SFE) gesture in all these postures is depicted in Figure 1.

Healthy participants were asked to perform the exercises in both sitting and standing positions to be consistent with the patients’ data. The number of repeats for each participant varied in the dataset. This was due to the limitations and complications of patients’ disorders, where some terminated the repetitions of a gesture in the middle of the experiment or decided not to perform a specific gesture. Some of the healthy participants also had different numbers of repetitions, but most of them completed 6 repeats in each standing and sitting position.

**Figure 2:**
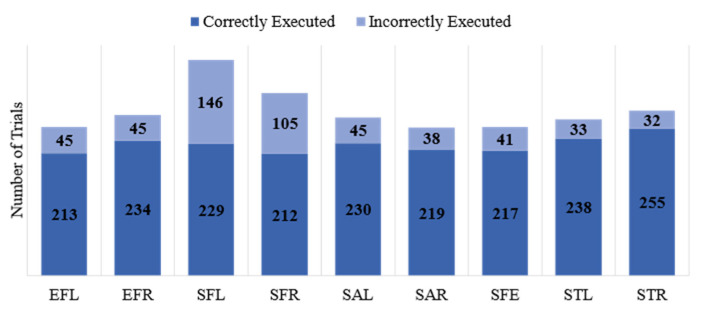
Number of trials for correctly and incorrectly executed exercises in the dataset.

Each repeat was labeled by physiotherapists as “Correctly Executed”, “Incorrectly Executed”, and “Poorly Executed”. In this paper, we considered the first two classes: “Correctly Executed” and “Incorrectly Executed”. We deemed the 12 “Poorly Executed” repetitions as “Incorrectly Executed”. As expected, 98% of the “Incorrectly Executed” labels belonged to the patients’ group. The numbers of correct and incorrect repetitions for each gesture are shown in Figure 2. This figure highlights the imbalanced nature of the dataset where about 80% of the exercises were performed correctly and only 20% were executed incorrectly.

We chose to use depth data instead of datasets containing conventional RGB videos to design a privacy-preserving system to be used at home. The proposed biofeedback system aims to be integrated into a design of tele-rehabilitation platform for in-home use. The privacy concerns of regular RGB cameras include exposing biometric markers such as a user’s face, soft biometric markers such as voice and gait, and non-biometric markers such as clothing and hairstyle [33]. The authors in [33] also showed that the compliance of the users to use the platform decreases when visual information is transmitted, processed and used. One suggested way to address these concerns is to use devices that provide depth footage instead [34,35,36]. The depth frames provide additional depth information while protecting the user’s privacy. It is also worth mentioning that the proposed system is compatible to use with any type of time-of-flight (ToF) sensor that provides depth footage, namely, Light Detection and Ranging (LiDAR), the Orbbec Astra Depth Camera, RGB-D camera, or Kinect.

For further details about the dataset, please refer to [31] by Miron et al.

### 3.2. Methodology

The main objective of this study is to automatically detect if the user performs the exercises correctly or incorrectly. In the first stage, we developed an activity recognition model which was trained on all “Correctly Executed” gestures. We applied a pre-trained model proposed by Carreira et al. [37] to classify the videos of participants performing all 9 gestures. This state-of-the-art network, called inflated 3D ConvNets (I3D), was trained on the Kinetics dataset [37], consisting of 400 different human actions such as person actions (singular), person—object actions, and person–person actions. An accuracy of 74.1% was achieved with the RGB data from the Kinects dataset, and an accuracy of 97.9% and 96.9% with UCF-101 [38] and HMDB-51 [39] data, respectively.

The I3D model was designed based on the Inception-v1 using batch normalization [40] with inflating filters and pooling kernels into 3D. The 3D model obtains videos of size 13 × 200 × 200 as the input, where 13 is the number of frames in the video and 200 × 200 is the resolution of each frame. Subsequently, in each layer the model extracts the features from videos using 3D convolutional kernels and 3D max pooling. The size of the convolutional kernels used in the sequence is different. The max pooling is used to prevent the over-fitting of the model by providing an abstract of the feature map. Eventually, a SoftMax activation function is used, and the scores are output. For deciding on the label of each video based on the predicted scores, an argument of the maxima (argmax) is calculated. In other words, the corresponding label to the highest score is considered to be the predicted label.

To recognize and classify the executed exercises, a categorical cross entropy was employed as our loss function, as follows:

(1) $$f\left(x\right)=-\frac{1}{N}\overset{N}{\underset{i=1}{\sum }}\overset{C}{\underset{c=1}{\sum }}1_{y_{i}\in C_{c}}\log\left(p_{model}\left[y_{i}\in C_{c}\right]\right)$$

where *i* iterates over *N* observations (the training sample size), *c* iterates over the number of categories (classes), the term $1_{y_{i}\in C_{c}}$ is the indicator function of the *i*th observation belonging to the *c*th category, and the log of probability is the logarithm of the probability predicted by the model for the *i*th observation belonging to the *c*th category. The goal was to minimize this loss function during the training phase. The Adam optimizer [41] was used, which uses estimations of the first moment and the second moment of the gradient to adapt the learning rate for each weight of the neural network. A dropout layer and early terminating criteria mechanisms are used to prevent overfitting of the model. As a result, if the loss function stops improving after a pre-determined number of epochs, also known as patience, the training process will automatically stop. The patience value was set to 15 epochs for our model.

**Figure 3:**
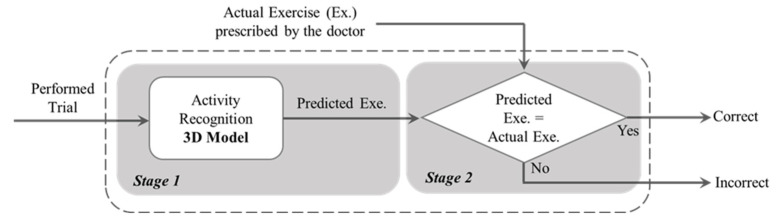
Schematic of the two-stage model for classifying the correct and incorrect gestures.

The models were validated based on both 10-Fold and LOSO cross validations. We conducted a grid search to find the best hyper-parameters such as batch size, learning rate, and number of epochs. The highest performance was achieved with a batch size and number of epochs of 64, and a learning rate of 0.0001 for both 10-Fold and LOSO. In order to create the binary architecture of our final model, in the second stage, we compared the output of the activity recognition model with the already known label. If the model correctly recognizes the activity type, it is labeled as a “Correctly Executed” exercise in the final output. Otherwise, the final output will show an “Incorrectly Executed” label. Figure 3 is a schematic representation of our architecture implemented in this study. This is based on this assumption that, if an exercise was misclassified by the first model, it is likely that this exercise was not performed correctly. We made this assumption based on the fact that, in the first stage, our model was pre-trained on a very large dataset of various video actions.

## 4. Results

**Figure 4:**
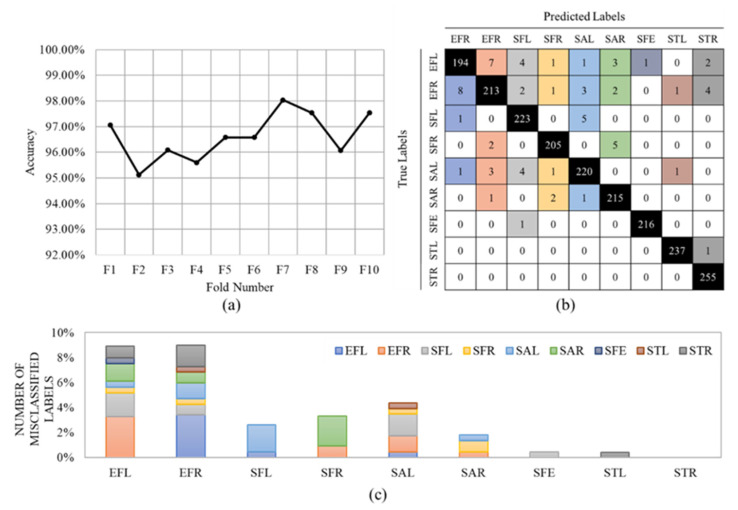
(**a**) The accuracy values per fold in 10-Fold, (**b**) the final confusion matrix, and (**c**) the total number of misclassified labels in 10-Fold. The colors in the confusion matrix (**b**) determine the class of the misclassified label in (**c**).

The proposed model was validated using both 10-Fold and LOSO cross validation. We calculated the classification performance using Equations (2)–(4), as follows:

(2) $$Accuracy=\frac{T_{P}+T_{N}}{T_{P}+T_{N}+F_{N}+F_{P}}\;,\;Precision=\frac{T_{P}}{T_{P}+F_{P}}$$

(3) $$Recall=\frac{T_{P}}{T_{P}+F_{N}}\;,\;Specificity=\frac{T_{N}}{T_{N}+F_{P}}$$

(4) $$F1\;Score=2\times \frac{Precision.Recall}{Precision+Recall}$$

where *T_P_*, *T_N_*, *F_P_*, and *F_N_* denote true positives, true negatives, false positives, and false negatives, respectively. In our application, the “Incorrectly executed” class is assumed as the positive class and “Correctly executed” as the negative class. A high number of true positives (correctly identifying the correct movements) and a low number of false negatives (correctly identifying the incorrect movements) are essential in our application. Given that the recall is of high importance in our problem, Figure 4a shows the accuracy values of the first stage for all 10 folds, separately.

An average accuracy of 96.62% ± 0.88% was achieved. The confusion matrix is shown in Figure 4b. The results show that our CNN model could classify all nine exercises with very high accuracy. The misclassified data are reported in Figure 4c. For example, this figure shows that the Side Tab (ST) gestures (Left and Right) had the lowest misclassification, whereas the Elbow Flexion (EF) gestures provided the highest misclassification. The high detection accuracy for the STL and STR might be because these two exercises were the only two lower-limb gestures and, therefore, the model could perfectly distinguish them. EFR and EFL exercises were misclassified 21 (9%) and 19 (8%) times. Most of the misclassified data belong to the same exercise but on the other side of the body.

**Figure 5:**
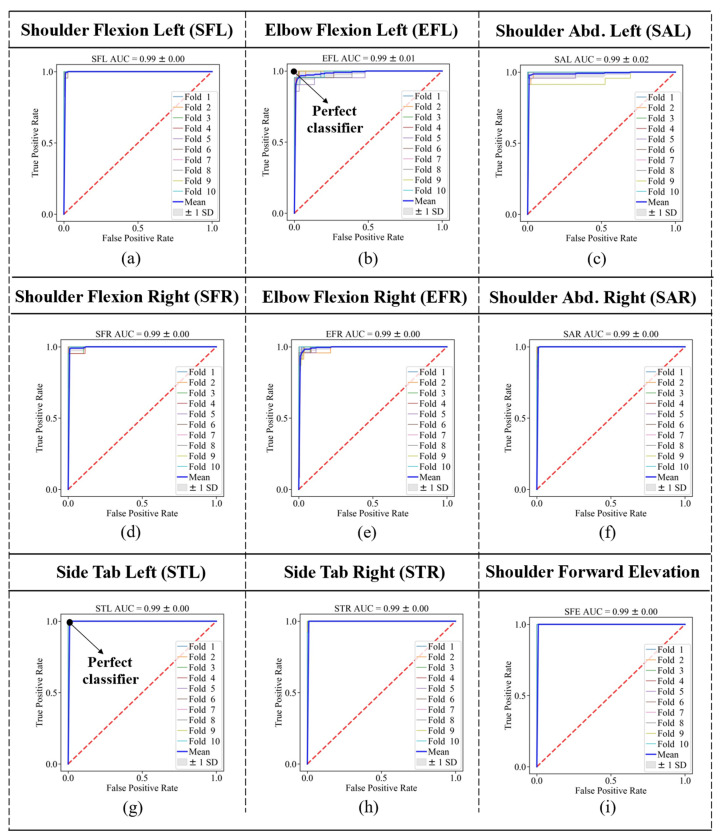
ROC curves considering 10-Fold cross validation for gestures (**a**) SFL, (**b**) EFL, (**c**) SAL, (**d**) SFR, (**e**) EFR, (**f**) SAR, (**g**) STL, (**h**) STR, and (**i**) SFE.

We also computed the AUC with the trapezoidal rule, using Equation (5), as follows [42]:

(5) $$f\left(x\right)=\overset{\;}{\underset{i}{\sum }}\frac{1}{2}\left(TPR_{i}+TPR_{i-1}\right)\left(FPR_{i}+FPR_{i-1}\right),$$

where *TPR* refers to the true positive rates and *FPR* refers to the false positive rates. AUC can achieve a maximum amount of 1, and the higher the AUC, the better the classifier. Receiver Operating Characteristic (ROC) curves are also shown for each gesture (class 1) versus the other eight gestures (class 2) in Figure 5.

**Figure 6:**
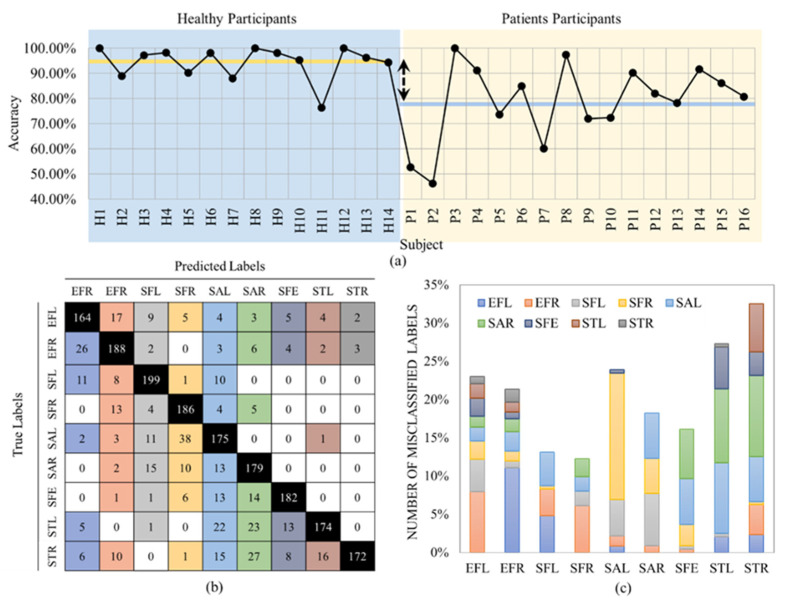
(**a**) Accuracy per subject for the LOSO model and the average accuracy in orange, (**b**) the final confusion matrix, and (**c**) the misclassification rate. The colors in the confusion matrix (**b**) determine the class of the misclassified label in (**c**).

The bold blue line in each graph shows the average of all 10 folds for that specific gesture. This figure also confirmed that the two lower limb exercises, STL and STR, provided the best ROC curves (perfect classifier); however, the EFL and EFR provided maximum distances to the perfect classifier (0,1), as shown in Figure 6c. The average ROC AUC achieved was 0.99 considering all gestures.

The LOSO cross validation for the exercise recognition stage resulted in an average accuracy of 86.04% ± 0.14%. Figure 6a shows the accuracy values per subject. Figure 6b,c show the confusion matrix and misclassified labels per exercise, respectively.

**Figure 7:**
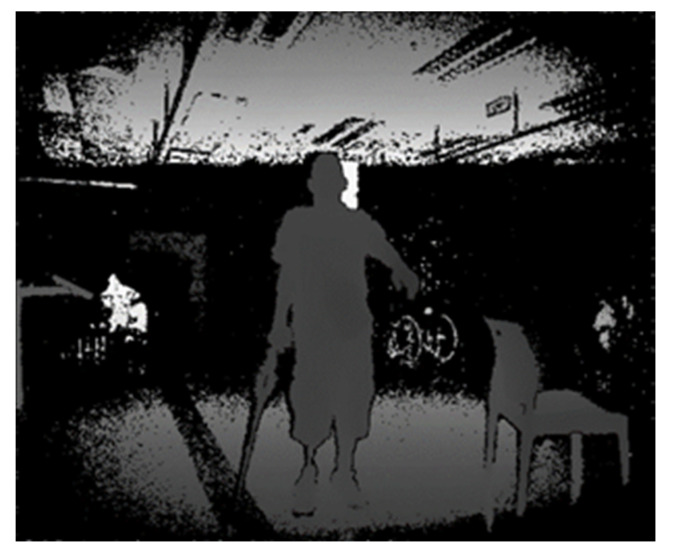
An example of SFL gesture performed by P2 holding a crutch.

As expected, the average accuracy was higher for healthy participants compared to the patient population with 94.30% ± 0.06% (yellow line in Figure 6a vs. 78.73% ± 0.15% (blue line in Figure 6a). This is because the healthy participants were able to perform the exercises almost perfectly; however, the patients were not able to exactly follow the patterns of the exercises due to the limited range of motion and pain that also resulted in higher standard deviation. Predictably, in LOSO, the number of misclassified labels was significantly higher than the 10-Fold cross validation. The accuracy rate decreased by about 10% compared to 10-Fold validation. The main reason is that different subjects performed the gestures in different postures. For example, subject P2, who had the least accuracy among all subjects (Figure 6a), was the only one that held a crutch during the performance of the trials, as shown in Figure 7.

In addition, unlike 10-Fold, STR and STL provided the highest misclassification rates in LOSO validation. This is also because the participants performed the exercises in different postures such as sitting on a chair/wheelchair or standing. These different lower body positions mostly affected the recognition of the two lower limb gestures.

**Figure 8:**
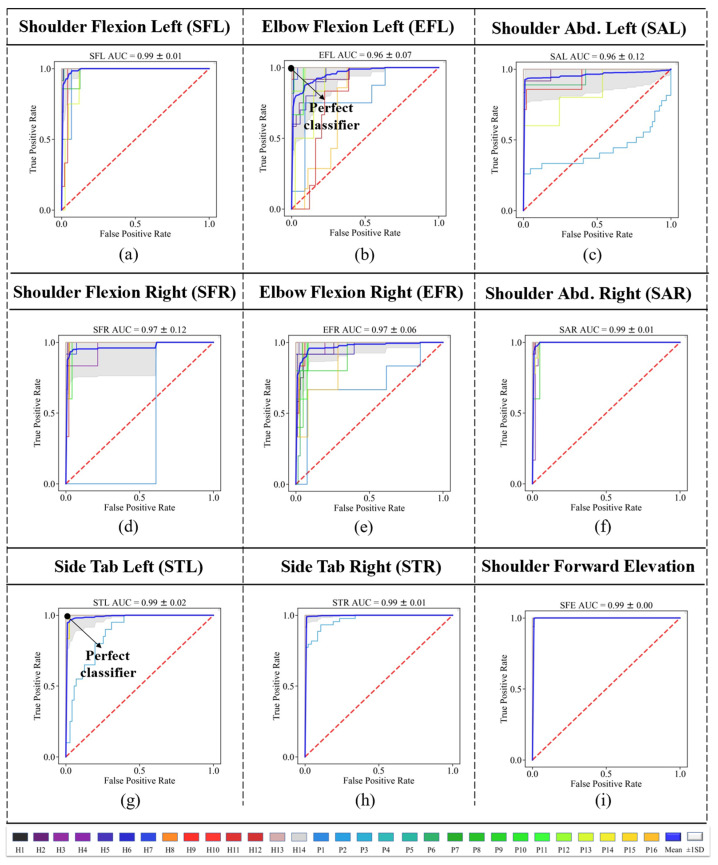
ROC curves for all subjects for gestures (**a**) SFL, (**b**) EFL, (**c**) SAL, (**d**) SFR, (**e**) EFR, (**f**) SAR, (**g**) STL, (**h**) STR, and (**i**) SFE. The mean curve is drawn in blue.

We also obtained the ROC curves for the LOSO approach, as depicted in Figure 8. The results of ROC show that SFE had the best classifier among all gestures. Participant P2 had the classifier with the lowest performance among all the participants.

**Figure 9:**
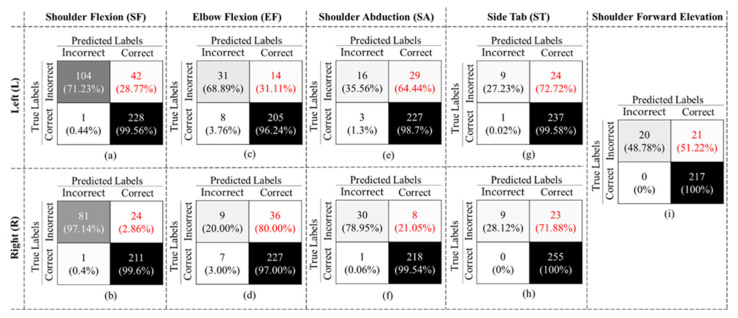
Confusion matrix for models (**a**) SFL, (**b**) EFL, (**c**) SAL, (**d**) SFR, (**e**) EFR, (**f**) SAR, (**g**) STL, (**h**) STR, and (**i**) SFE obtained using 10-Fold cross validation.

After training the exercise recognition model on “Correctly Executed” gestures in the first stage, we implemented the second stage, where we detected if the exercise was performed “Correctly” or “Incorrectly” to build our final binary structure, as shown in Figure 3. The confusion matrices in Figure 9 are the results of our 10-Fold cross validation.

**Figure 10:**
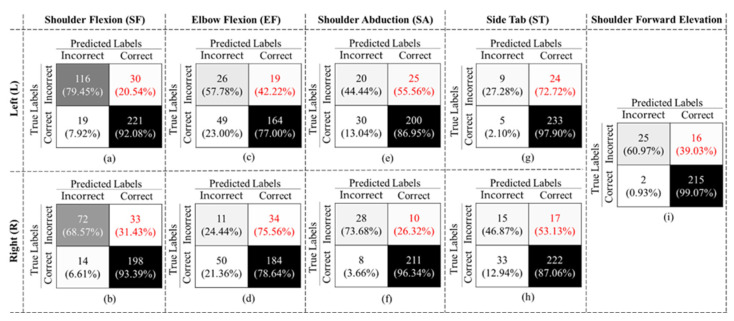
Confusion matrix for models (**a**) SFL, (**b**) EFL, (**c**) SAL, (**d**) SFR, (**e**) EFR, (**f**) SAR, (**g**) STL, (**h**) STR, and (**i**) SFE obtained using the LOSO approach.

The final binary model achieved an overall accuracy of 90.57% ± 9.17, an F1-Score of 71.78% ± 5.68, and specificity and recall of 93.35% ± 5.20 and 58.30% ± 23.71, respectively, considering all gestures. As shown in Figure 10, in all cases, the false negative rates (highlighted in red font) are larger than the false positive rates, which resulted in a low recall value. We also evaluated our binary classifier using the LOSO approach. The confusion matrices of all nine gestures are displayed in Figure 10.

As expected, the performance of the LOSO model was slightly lower than the 10-Fold validation. Table 3 also summarizes the results. The value in each row represents the average of performance of all 30 subjects for each gesture. As shown in this table, all gestures achieved an accuracy rate of more than 69%, with the highest accuracy of 93.02% for SFE. The mean accuracy and F1-Score across all gestures and subjects were 83.78% ± 7.63 and 60.64% ± 25.14, respectively. Comparing our results to previous studies that used similar Kinect technology in [21,30], we could achieve the highest performance in both activity recognition and binary classification.

Table 4 summarizes the results of the 10-Fold and LOSO cross validations. It is worth noting that although the overall performance decreased in LOSO, the total number of false negatives was less than 10-Fold. In other words, LOSO could better recognize the incorrectly executed gestures than the 10-Fold method.

We compared the results of our study to the most recent related works considering the technology, number of activities and method of assessing exercise performance quality. We have included vision-based studies in this comparison. Table 5 summarizes the performance of these models.

Table 5 contains the recent works relevant to our study, including exercise recognition (ER) and exercise assessment (EA). For the exercise assessment, which is the primary purpose of our study, we found two vision-based studies: [21,44]. Both studies provided roughly similar performance to our proposed model, whereas our dataset consisted of more types of exercises. In addition, these studies did not report any performance evaluation for LOSO, which often provides lower performance compared to methods such as k-fold.

## 5. Limitations of the Study

Although vision-based systems are useful contactless technology, their outputs may be subject to occlusion or poor performance under various light conditions [45]. In addition, the accuracy of the algorithms is limited to the resolution and the distance of the camera to the subject.

The major limitation of this study is the small sample size of “Incorrectly Executed” gestures. This prevented the model from properly learning the differences between the two classes. In the future, we plan to collect data from more patients to perform transfer learning and create a more robust biofeedback model.

The second limitation of our study is in the determination of the correctness of an exercise in the labeling phase. Upon checking the misclassified videos, we discovered that many of these trials were very close to the correct gestures. It is critical to have precise labels for training a deep learning model. Different clinicians may label a single trial differently, especially for the trials that are very close to the correct pattern. Therefore, a standard procedure is required to ensure that all labels are consistent from different viewpoints. In the future, we plan to prepare a list of criteria to detect if an exercise was performed correctly or incorrectly with clinicians’ input. This list can be further used in our labeling phase, where different numbers of clinicians are able to rate these criteria and the final labels can be achieved with higher confidence. In addition, we will perform a statistical analysis to investigate if there are any significant differences among the clinicians’ rates and labels.

Given the low number of available lower extremity exercises in our dataset, in future, we plan to add more exercises that can cover this limitation and conduct further research with greater focus on lower limb exercises.

In future, we will evaluate our proposed models considering other skeleton tracking/pose estimation algorithms, such as MediaPipe and MoveNet, in order to obtain the best pre-train models for our biofeedback system.

## 6. Conclusions

In this paper, we presented a novel state-of-the-art 3D inflated CNN model to detect the correct and incorrect exercises performed by the patients and provide them with feedback to refine their movements. We used an open dataset to design and validate our model. Our model could achieve 90.57% accuracy and a 71.78% F1-Score for 10-Fold validation. Moreover, for the LOSO validation approach, our model could achieve 83.78% and 60.64% accuracy and F1-Score, respectively. The proposed model can be readily used for assessing any exercise with high accuracy at home, which can reduce potential costs, time, and the risk of infectious disease transmission. We are expanding this platform for a better understanding of the rehab exercises by extracting features such as the inter-relationships of different parts of the body, such as muscles. Consequently, therapists can better improve the exercises remotely.

## Figures and Tables

**Table 1:** Summary of vision-based approaches reviewed in this study.

Ref.	Sub.#	Problem	Method	Performance/Results	
[18]	78	Designing an automatic assessment in tele-rehab	DMDN	RMSE ^1^: 11.99%	
[20]	52	Predicting the rehabilitation outcome and classifying healthy and MS participants	KNN SVM	RMSE: 6.1%	
				Classification:	
				Acc. ^2^: 91.7% Acc.: 88.0%	AUC ^3^: 96% AUC: 93%
[30]	23	Estimating the Brunnstrom scale	RF SVM Hybrid model	Acc.: 58.8% Acc.: 55.7% Acc.: 84.1%	
[21]	35	A two-phase human action understanding algorithm	Hidden Markov SVM	Acc. for feedback: 93.5% Acc. for task recommendation: 90.47%	
[26]	1	Detecting static posture and falls	NN	Acc.: 96%, Pre. ^4^: 95%, Rec. ^5^: 97% and F1-Score: 96%	
[27]	20	Rehabilitation exercise recognition	3-layer CNN-LSTM	Acc.: 91.3%	
[28]	2	Shoulder angle estimation	NN	Acc.: 67.04%	
[29]	8	Rehabilitation exercise recognition	MDNN	Acc.: 67.04%	

^1^ RMSE: root mean square error, ^2^ accuracy, ^3^ area under curve, ^4^ precision, ^5^ recall.

**Table 2:** Gestures indexes and descriptions.

Index	Gesture Name	Description
0	Elbow Flexion Left (EFL)	Flexion and extension of the left elbow joint
1	Elbow Flexion Right (EFR)	Flexion and extension of the right elbow joint
2	Shoulder Flexion Left (SFL)	Flexion and extension of the left shoulder while the arm is kept straight in front of the body
3	Shoulder Flexion Right (SFR)	Flexion and extension of the right shoulder while the arm is kept straight in front of the body
4	Shoulder Abduction Left (SAL)	Maintaining the arm straight, the left arm is raised away from the side of the body
5	Shoulder Abduction Right (SAR)	Maintaining the arm straight, the right arm is raised away from the side of the body
6	Shoulder Forward Elevation (SFE)	Holding hands clasped together in front of the body, maintaining the arms in a straight position, raise the arms above the head while keeping elbows straight
7	Side Tap Left (STL)	Moving the left leg to the left side and back while maintaining balance
8	Side Tap Right (STR)	Moving the right leg to the right side and back while maintaining balance

**Table 3:** LOSO results for all gestures.

Gesture	Accuracy (%)	Precision (%)	F1-Score (%)	Specificity (%)	Recall (%)
EFL	73.64 ± 34.38	34.67 ± 42.99	43.33 ± 43.49	77.00 ± 33.75	57.78 ± 27.59
EFR	69.89 ± 32.25	18.03 ± 46.76	20.75 ± 45.23	78.63 ± 29.25	24.44 ± 39.53
SFL	86.93 ± 19.66	85.93 ± 38.43	82.56 ± 38.15	91.70 ± 18.42	79.45 ± 26.96
SFR	85.17 ± 19.54	68.57 ± 40.54	75.39 ± 41.57	85.71 ± 23.86	83.72 ± 23.01
SAL	80.00 ± 24.65	40.00 ± 35.95	42.11 ± 43.29	86.96 ± 16.61	44.44 ± 40.00
SAR	93.00 ± 25.82	77.78 ± 34.55	75.68 ± 37.24	96.35 ± 18.85	73.68 ± 22.64
SFE	93.02 ± 16.87	92.59 ± 19.79	73.53 ± 29.18	99.08 ± 2.25	60.98 ± 27.19
STL	89.30 ± 23.72	64.29 ± 24.94	38.30 ± 24.97	97.90 ± 4.59	27.27 ± 34.91
STR	82.58 ± 25.32	31.25 ± 33.12	37.50 ± 41.82	87.06 ± 17.87	46.88 ± 32.96
**Overall**	**83.78 ± 7.63**	**60.53 ± 25.14**	**60.64 ± 21.3**	**89.74 ± 7.53**	**60.75 ± 20.25**

**Table 4:** LOSO and 10-Fold results for final binary classification.

Accuracy (%)		Precision (%)		F1-Score (%)		Specificity (%)		Recall (%)	
10-Fold	LOSO	10-Fold	LOSO	10-Fold	LOSO	10-Fold	LOSO	10-Fold	LOSO
90.57%	83.78%	93.35%	60.53%	71.78%	60.64%	98.93%	89.74%	58.30%	60.75%

**Table 5:** A comparison of our work to the most recent similar works.

Ref. #	ER ^1^ Accuracy		EA ^2^ Accuracy		EA F1-Score		Device	Activities	Feedback
	LOSO	Other	LOSO	Other	LOSO	Other			
[27]	-	91.3%	-	-	-	-	RGB	7	NA
[18]	-	-	-	RMSE:0.12	-	-	Kinect	5	10 Level Numerical
[21]	-	98.13%	-	93.5%	-	-	Kinect	3	Binary
[43]	-	-	-	92.33%	-	-	Kinect	4	Numerical into Binary
**Proposed**	**86.04%**	**96.62%**	**83.78%**	**90.57%**	**60.64%**	**71.78%**	**Kinect**	**9**	**Binary**

^1^ ER: exercise recognition, ^2^ EA: exercise assessment.

## Data Availability

The dataset by Miron et al. [29] is available at: https://zenodo.org/record/4610859 (accessed on 3 August 2021).
